# Supplementary material for: Targeting FANCM by antisense oligonucleotides in ALT-positive cancers
Source: Mol Ther Nucleic Acids. 2025 Feb 20;36(2):102492. doi: 10.1016/j.omtn.2025.102492 (PMC11930073; doi:10.1016/j.omtn.2025.102492)
Supplement: Document S1. Figures S1–S4 [file mmc1.pdf]

OMTN, Volume *36*

Supplemental information

Targeting FANCM by antisense oligonucleotides in ALT-positive cancers

Galen Tieo, Natalie Bao Ying Lim, Kah Wai Lim, Peter Dröge, Anh Tuân Phan, and Maya Jeitany

Figure S1

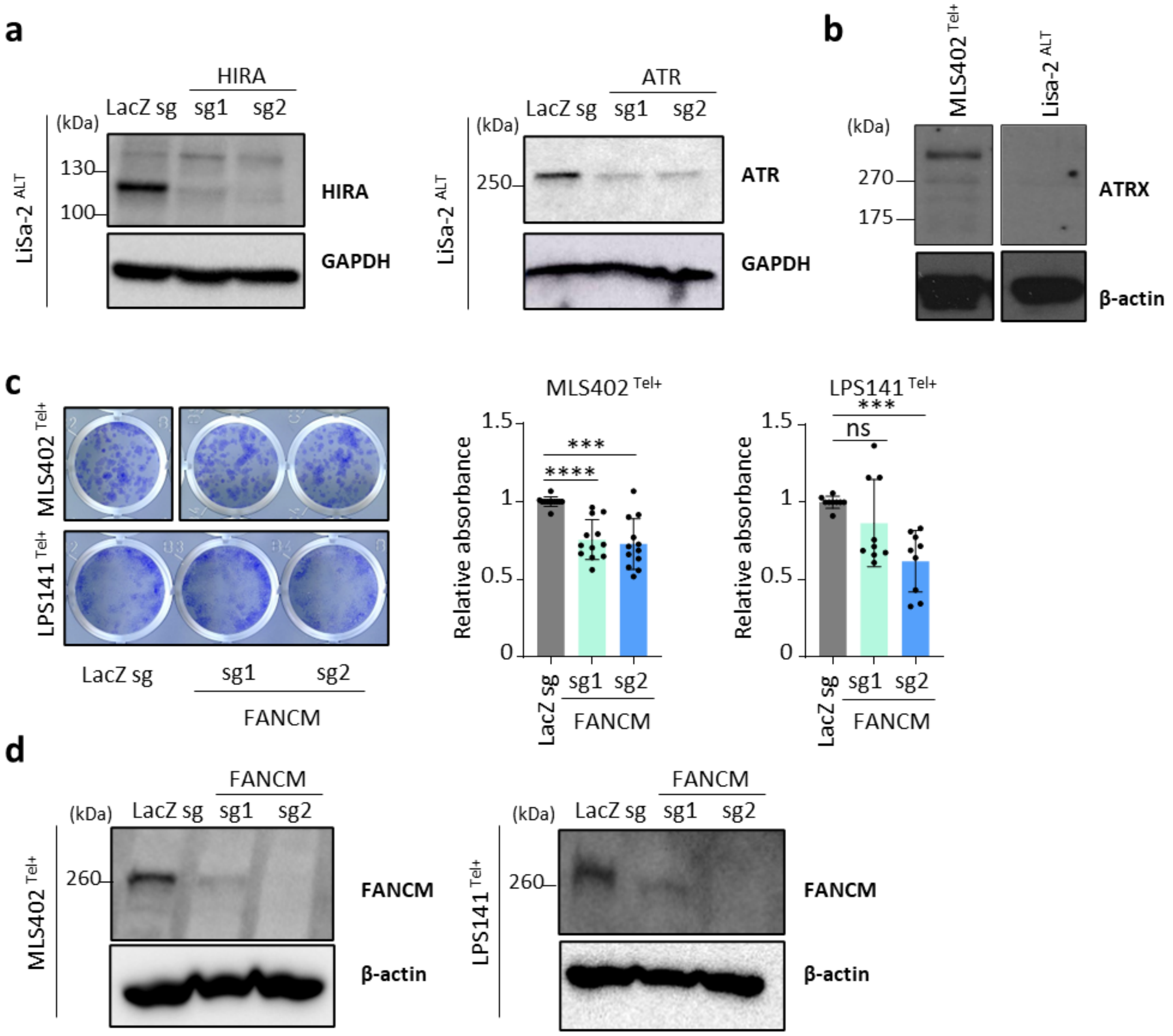

Figure S1: a) Western blots verifying depletion of HIRA or ATR in the corresponding CRISPR-Cas9 edited LiSa-2 cells, compared to LacZ sg control cells. **b)** Western blot showing expression levels of ATRX in MLS402 and LiSa-2 liposarcoma cells. **c)** Colony formation assays for MLS402 or LPS141 telomerase-positive cells, after FANCM depletion. The graphs represent the absorbance quantification (mean ± SD), after staining and lysing of the fixed colonies, relative to corresponding control cells (LacZ sg). Values are from four independent experiments with three biological replicates each for MLS402, and three independent experiments with three biological replicates for LPS141. (***p<0.001, ****p<0.0001, ns = not

significant, as determined by two-tailed paired t-test). **d)** Western blot verifying the reduction of FANCM protein levels in MLS402 and LPS141 FANCM sg1 and sg2 cells. β-actin and GAPDH serve as loading controls.

Figure S2

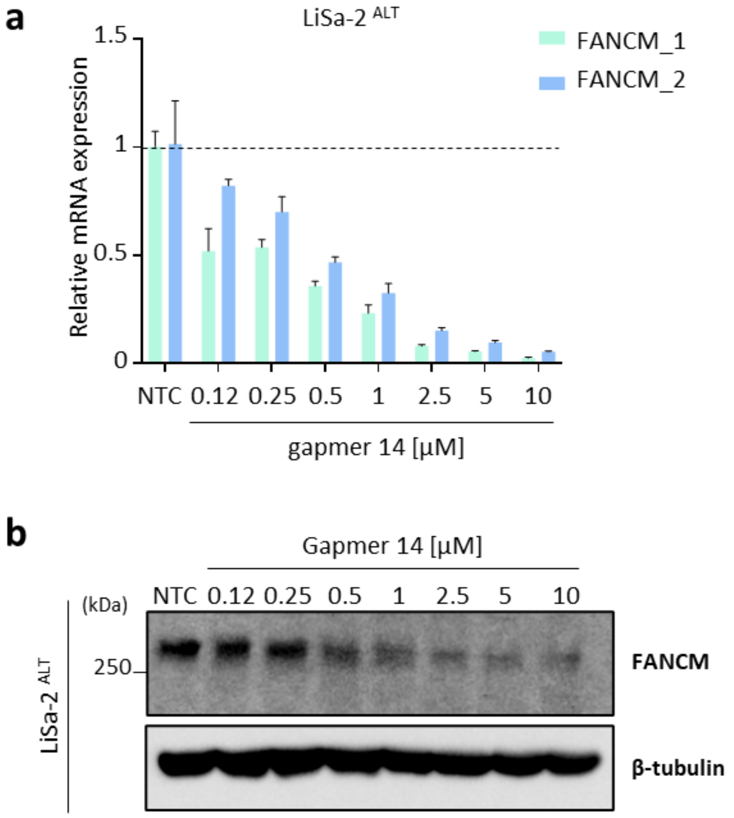

Figure S2: a) FANCM relative mRNA levels detected by qRT-PCR in LiSa-2 cells treated with different concentrations of gapmer 14. NTC gapmer was used at 10 µM. Means ± SD are calculated from three biological replicates. **b)** Western blot assessing FANCM protein levels in LiSa-2 cells treated with similar conditions as in (a). β-tubulin serves as a loading control.

Figure S3

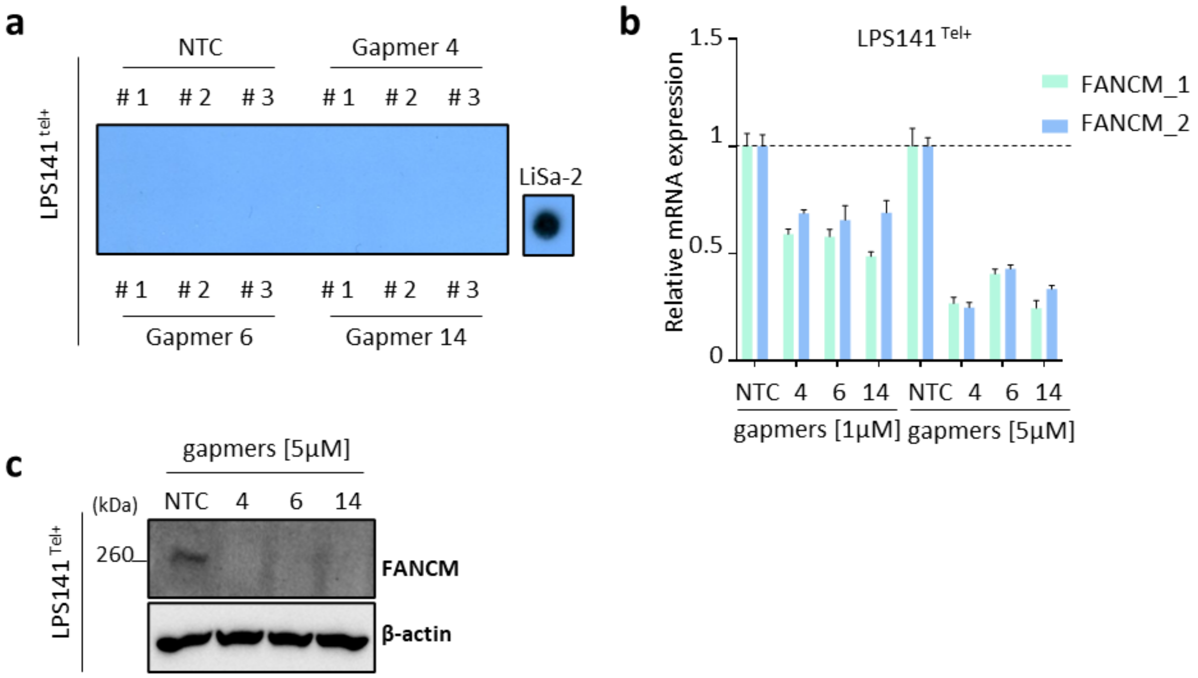

Figure S3: a) Telomeric C-circle assays on LPS141 telomerase-positive cells after 72 hours of treatment with 5 µM of control (NTC) or FANCM-targeting gapmers 4, 6 or 14 (three biological replicates each). LiSa-2 is shown as a positive control. **b)** FANCM mRNA levels detected by qRT-PCR in LPS141 treated with either 1 or 5 µM of indicated gapmers. Values (mean ± SD) are shown relative to NTC control and are calculated from three biological replicates. **c)** Western blot detecting FANCM in LPS141 treated with gapmers for 72 hours. β-actin is a loading control.

Figure S4

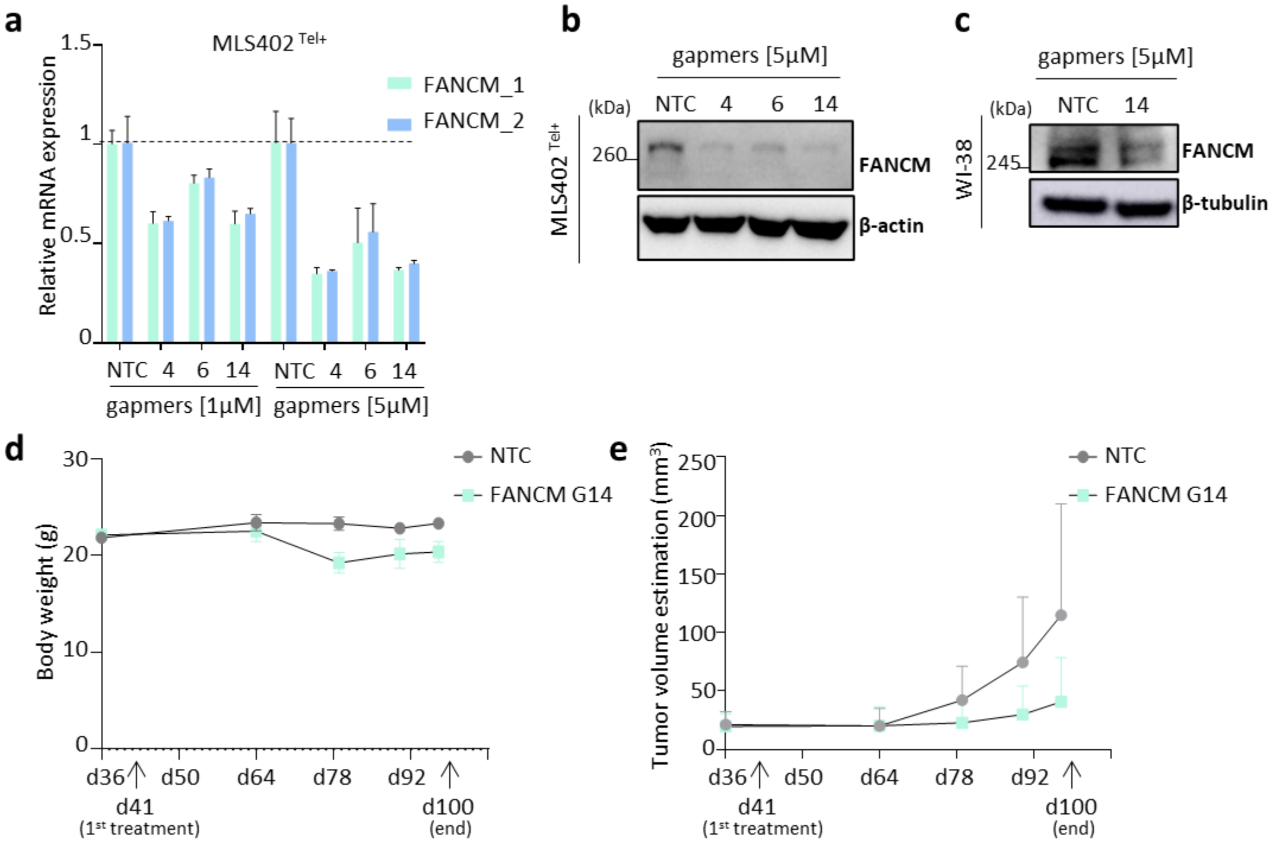

Figure S4: a) qRT-PCR detection of FANCM expression in MLS402 cells treated with indicated gapmers for 48 hours. Relative mean (± SD) of three biological replicates is shown for each condition. **b-c)** Western blot analyses showing reduction of FANCM protein levels in MLS402 cells treated with 5 µM of gapmers 4, 6 or 14 for 72 hours (b), and in WI-38 cells treated with gapmer 14 for 72 hours (c). β-actin serves as a loading control. **d-e)** Follow-up of body weight (d) and tumor volume (e) of mice from the experiment in 4g-h. Values are mean (± SD) body weight of all animals (d) or tumor volumes (e).
