# Supplementary material for: Targeting FANCM by antisense oligonucleotides in ALT-positive cancers
Source: Mol Ther Nucleic Acids. 2025 Feb 20;36(2):102492. doi: 10.1016/j.omtn.2025.102492 (PMC11930073; doi:10.1016/j.omtn.2025.102492)
Supplement: Document S2. Article plus supplemental information [file mmc2.pdf]

Molecular Therapy
Nucleic Acids
Original Article

Targeting FANCM by antisense oligonucleotides in ALT-positive cancers

Galen Tieo,[1,3] Natalie Bao Ying Lim,[2,3] Kah Wai Lim,[2] Peter Dröge,[1] Anh Tuân Phan,[2] and Maya Jeitany[1]

[1]School of Biological Sciences, Nanyang Technological University, Singapore 637551, Singapore; [2]School of Physical and Mathematical Sciences, Nanyang Technological University, Singapore 637371, Singapore

Effective therapies for cancers relying on the alternative lengthening of telomeres (ALT) mechanisms are still needed. Here, using CRISPR-Cas9 strategies, we validate FANCM (Fanconi anemia complementation group M) as a crucial target for ALT-associated cancers and demonstrate its importance in both *in vitro* and *in vivo* models. We further explore the use of antisense oligonucleotides (ASOs), specifically gapmers, to target FANCM mRNA. We designed and screened several gapmers, identifying effective candidates that potently reduced FANCM expression, which led to an increased ALT activity and telomeric dysfunction, concomitant with a reduced viability of ALT-positive cancer cells. Notably, gapmer 14, one of the identified ASOs, significantly impaired the viability of ALT cells and reduced tumor growth in an ALT-positive liposarcoma xenograft model, highlighting its therapeutic potential. These findings suggest that FANCM-targeting ASOs could represent a promising effective strategy for treating ALT-positive cancers.

INTRODUCTION

To avoid replicative senescence and maintain the telomeric ends of their chromosomes, a subset of cancers utilize homology-directed repair (HDR) mechanisms known as alternative lengthening of telomere (ALT).[1–3] ALT mechanisms are frequently detected in some tumor types such as sarcomas and glioblastomas, therefore representing an appealing therapeutic opportunity for these cancers.[3] Several strategies to target ALT have been proposed in the past decade, including inhibition of ATR,[4] HIRA,[5] or the use of the kinase inhibitor ponatinib.[6] Despite this progress, ALT-centred therapies have not yet been clinically implemented.

In the absence of functional telomerase, ALT mechanisms involve various DNA replication and repair pathways to elongate and maintain the telomeres of these cancer cells, including homologous recombination (HR)[7] or break-induced replication (BIR).[8] ALT cells use these mechanisms to repair telomeric damage and resolve replicative stress, both inherently found at ALT telomeres, allowing efficient telomere replication and telomere synthesis.[9]

One of the important proteins contributing to preserving telomeres of ALT cells is FANCM (Fanconi anemia complementation group M).[10] FANCM is a component of the Fanconi anemia core complex. FANCM possesses a translocase activity and can participate in several DNA repair and replication stress response functions, including promoting replication fork reversal, restricting recombination, and resolving stalled replication at interstrand crosslinks (ICL).[11,12] In ALT cells, FANCM is an important regulator of telomere integrity. Depletion of FANCM provokes aberrant ALT activity, demonstrated by an increase of several ALT-specific features, and an exacerbation of break-induced telomere synthesis. This unrestrained ALT activity was found to be detrimental to ALT cell viability; FANCM-deficient cells had a lower potential to form colonies in clonogenic assays.[13] FANCM capacity to suppress ALT was shown to be mediated by its DNA translocase and replication fork remodeling activities, as well as its functional interaction with the BLM-TOP3A-RMI (BTR) complex.[13,14] Disruption of the FANCM-BTR complex using a small molecule inhibitor of the MM2-RMI interaction, PIP-199, could specifically inhibit the viability of ALT cells, as shown in U2OS, SAOS-2, and GM847 ALT cells.[13] The importance of FANCM in controlling ALT mechanisms was also validated by other studies. Silva et al. showed that FANCM, in an ATPase/translocase-dependent manner, resolves replication stress at ALT telomeres by unwinding telomeric RNA:DNA hybrids (R-loops) and subsequently limiting an uncontrolled BLM activity at ALT telomeres.[14] FANCM suppression with short interference RNAs (siRNAs) in several ALT cell lines, but not in telomerase-positive ones, led to a decrease in clonogenic potential and an aberrant accumulation in G2/M phase.[14] Similarly, Pan et al. showed that FANCM co-operates with BLM and BRCA1 to resolve replication stress at ALT telomeres, and that co-depleting FANCM and either BLM or BRCA1 is lethal to ALT cells.[10]

Disrupting the interaction between RMI (from the BTR complex) and FANCM (on the MM2 peptide) has been tested as a potential anti-ALT strategy, including an ectopic expression of an MM2 peptide that acts as an inhibitor of this interaction.[13,15] Another compound,

Received 3 September 2024; accepted 17 February 2025;
https://doi.org/10.1016/j.omtn.2025.102492.

[3]These authors contributed equally

Correspondence: Anh Tuân Phan, School of Physical and Mathematical Sciences, Nanyang Technological University, Singapore 637371, Singapore.
E-mail: phantuan@ntu.edu.sg
Correspondence: Maya Jeitany, School of Biological Sciences, Nanyang Technological University, Singapore 637551, Singapore.
E-mail: maya.jeitany@gmail.com

Published by Elsevier Inc. on behalf of The American Society of Gene and Cell Therapy.
This is an open access article under the CC BY license (http://creativecommons.org/licenses/by/4.0/).

PIP-199, was identified through a high-throughput screening as an RMI-MM2 inhibitor.[16] As mentioned above, PIP-199 was shown to moderately alter ALT activity *in vitro*. However, the efficacy of this molecule was not tested *in vivo* and concerns were raised regarding its specificity and chemical stability.[17] Recently, Alcock et al. developed a competitive assay to allow screening for FANCM-RMI disruptors.[18] While other domains of FANCM could potentially be considered for drug development, such as those necessary for FANCM interaction with key proteins (e.g., with FAAP24) or those conferring FANCM its enzymatic activities (e.g., ATPase motor domain),[19] no small molecule has yet been fully characterized and validated as an FANCM functional inhibitor. Therefore, alternative therapeutic approaches for targeting FANCM are needed.

Antisense oligonucleotides (ASOs) are a rapidly emerging drug modality that has seen promising potential in the clinic.[20] ASOs engage specifically with target RNA through complementary Watson-Crick base pairing and since they function at the RNA level, lead compounds can be rationally designed based on genetic information, which could expedite the drug development process. Furthermore, ASOs have demonstrated the ability for efficient free uptake in certain cells, otherwise known as gymnosis, which leverages the inherent growth properties of the cells to facilitate the oligonucleotide uptake.[21] Gapmers are one class of ASOs that engage the intracellular ribonuclease H1 (RNase H1) to reduce the target mRNA levels. They are composed of a central DNA segment flanked by two modified segments for enhanced binding affinity. Following pairing with RNA targets, DNA-RNA hetero-duplexes are formed and become substrates for RNase H1 that cleaves the RNA strand, hence leading to RNA degradation and a reduction in protein translation.[22–24]

In this study, we validated FANCM as a potent ALT-associated target, *in vitro* and *in vivo*, using a CRISPR-Cas9 strategy. We then designed and screened gapmer ASOs targeting FANCM mRNA. Potent candidates were identified and lead ASOs were proven to efficiently reduce both FANCM mRNA and protein levels. Our findings indicate that these FANCM ASOs alter ALT activity, highlighted by an increase in key features of ALT, including an accumulation of extrachromosomal telomeric C-circles as well as telomeric dysfunction. Identified ASOs were also shown to decrease specifically the viability of ALT-positive cancer cells. Importantly, these ASOs were not only effective *in vitro* but also demonstrated potency in limiting tumor growth *in vivo*. Overall, our study validates the essential role of FANCM for ALT cancers *in vivo* and proposes FANCM-targeting ASOs as an effective strategy for anti-ALT therapies.

RESULTS

FANCM depletion affects the viability of cells relying on alternative lengthening of telomeres

We assessed the importance of several reported targets for ALT cancer cells: FANCM (a protein of the Fanconi Anemia pathway that suppresses telomeric replication stress in ALT cells),[10,13,14,19,25,26] HIRA (a modulator of histone H3.3 deposition contributing to recombination activities at ALT telomeres),[5] and ATR (a regulator of recombination at ALT telomeres).[4] Using the CRISPR-Cas 9 system, we individually depleted each of these targets (Figures 1A and 1B) and validated the down-regulation of their protein levels (Figures 1C and S1A). We then assessed the survival potential of ATRX-deficient LiSa-2 ALT cancer cells (Figure S1B) in clonogenic assays (Figures 1A and 1B). Among the tested candidates, depletion of FANCM with two different guide RNAs (sg1 and sg2) led to a pronounced reduction in the clonogenic potential of LiSa-2 cells (Figures 1A–1C), compared with HIRA sgRNAs and ATR sg1 treatments, while the effect on two telomerase-positive cell lines was less pronounced (Figures S1C and S1D), as previously reported.[13,14] We also confirmed that the absence of FANCM increases ALT activity, evaluated by the levels of telomeric C-circles (Figure 1D). Importantly, *in vivo*, mice inoculated subcutaneously with LiSa-2 cells depleted for FANCM, had no or lower tumor burden, compared with mice injected with control cells (Figures 1E and 1F). These results further validate the role of FANCM in regulating ALT mechanisms and demonstrate its necessity for ALT cancer cell survival in a preclinical *in vivo* human xenograft model.

FANCM-specific ASOs potently reduce FANCM levels

To identify potent FANCM ASO candidates, we designed multiple fully phosphorothioated, locked nucleic acid (LNA) ASOs with a 3-10-3 gapmer configuration[22] (a middle segment formed of 10-mer DNA and flanked by 3-mer LNA on each side) (Figure 2A) targeting locations at the 5′ and 3′ regions of FANCM mRNA (Figure 2B). The phosphorothioate (PS) modification renders the backbone more resistant to nuclease degradation and therefore more stable in the biological system.[27] Furthermore, the PS modification enhances the binding affinity of ASOs to serum proteins, which is necessary for the distribution of ASOs to peripheral tissues and hence is important for its uptake. This protects the ASOs against a rapid clearance out of the body.[28]

The effectiveness of these ASOs in initiating RNase H1-mediated mRNA cleavage was examined in SAOS-2 ALT cells, using quantitative real-time PCR (qRT-PCR), following 48 h of ASO treatment at 1 μM (Figure 2C). The capacity of ASOs for free uptake, by gymnosis, eliminated the need to use transfectant reagents.[21] Relative levels of FANCM mRNA were compared with those in control cells treated with a non-targeting control (NTC) oligonucleotide comprising the same LNA gapmer chemistry. Within three distinct regions of FANCM mRNA, we identified at least three ASOs (gapmers 4, 6, and 14) that yielded >50% knockdown of FANCM mRNA (Figure 2C). The target region of gapmer 14 was further evaluated by designing gapmers 28, 29, and 30 that targeted sequences overlapping with that of gapmer 14. These gapmers did not improve the knockdown of FANCM mRNA (Figure 2D). Therefore, our subsequent testing focused on the three ASOs, gapmers 4, 6, and 14. Gapmers 4, 6, and 14 efficiently reduced FANCM mRNA levels in two other ALT cells lines, U2OS and LiSa-2, with gapmer 14 showing the most robust activity in U2OS cells (>90% knockdown of FANCM mRNA) (Figure 2E).

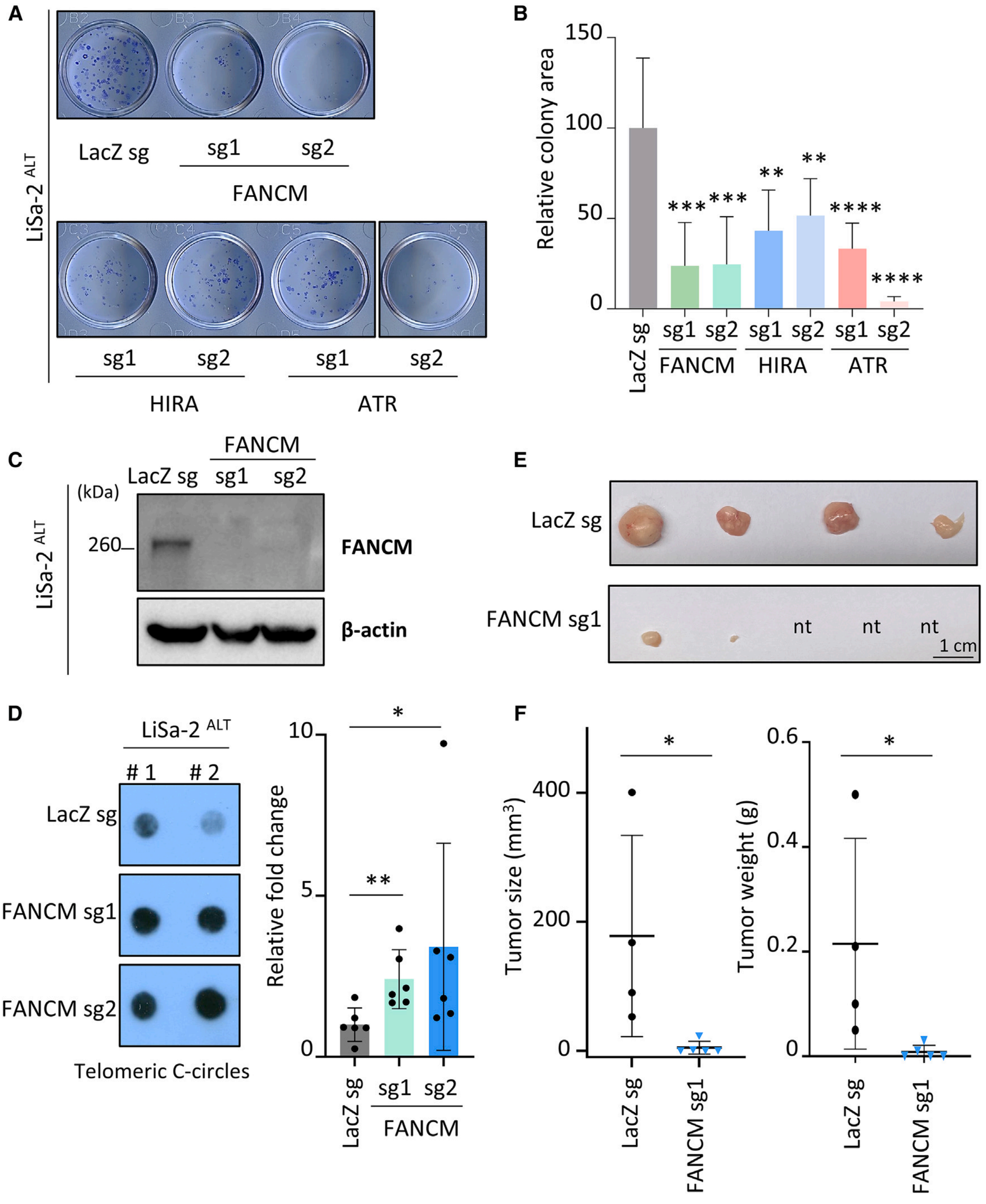

Figure 1. CRISPR-mediated depletion of FANCM affects ALT activity and ALT cell survival

(A) Colony formation assays on LiSa-2 cells transduced with CRISPR-Cas9 system and single guide RNAs (sg1 and sg2) targeting either FANCM, HIRA, ATR, or LacZ (as a control). (B) Quantification of relative colony area from experiments representatively shown in (A). Values are from at least five biological replicates from two independent experiments. **$p < 0.01$, ***$p < 0.001$, ****$p < 0.0001$, ns = not significant, as determined by two-tailed paired t test. (C) Western blot showing absence of FANCM proteins in FANCM-depleted LiSa-2 cells. β-actin is used as a loading control. (D) Telomeric C-circle assays in LiSa-2 cells lacking FANCM. The left pictures are examples of two replicates (#1, #2) of telomeric C-circles in each condition (LiSa-2 transduced with LacZ sg, FANCM sg1 or sg2). The right graph shows the quantified levels (mean ± SD) of telomeric C-circles in FANCM-depleted cells, relative to control cells (LacZ sg). Values are from six biological replicates from two independent experiments. (E) Pictures of LiSa-2 xenograft tumors extracted from mice at the endpoint of the *in vivo* experiment. Each tumor comes from one animal (nt = no tumor). (F) Graphs depicting the size (left) and weight (right) (mean ± SD) of the tumors visualized in (E). *$p < 0.05$, **$p < 0.01$, ns = not significant, as determined by two-tailed Mann-Whitney test.

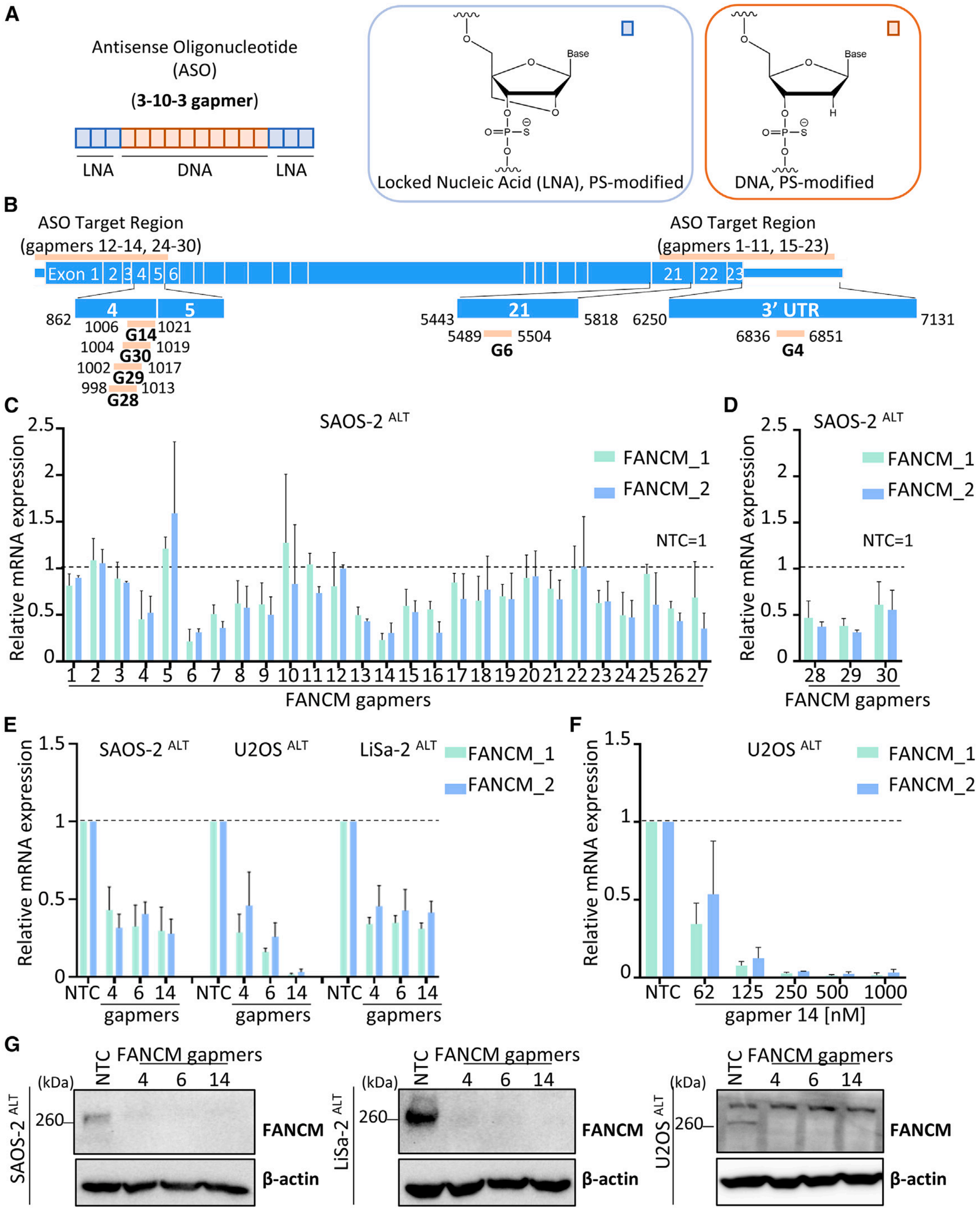

Figure 2. FANCM-targeting gapmers potently reduce FANCM levels

(A) Schematic of the composition of ASOs used in this study, with a 3-10-3 (LNA-DNA-LNA) configuration. (B) Schematic of FANCM mRNA depicting the exons, the 5′ and 3′ UTR, as well as ASO target regions for indicated gapmers. Specific binding regions are shown for gapmers 4, 6, 14, 28, 29, and 30. (C and D) FANCM mRNA levels in SAOS-2 cells treated with different gapmers. Levels are detected by quantitative real-time PCR (qRT-PCR) using two different sets of primers (FANCM_1 and FANCM_2) and means (±SD) are represented relatively to FANCM levels in SAOS-2 cells treated with a non-targeting control gapmer (NTC). Values are from at least two biological replicates. (E) FANCM levels (mean ± SD) detected by quantitative real-time PCR (qRT-PCR) in SAOS-2, U2OS, and LiSa-2 treated for 48 h with 1 μM of NTC gapmer, or FANCM-targeting gapmers 4, 6, or 14. Values are from three biological replicates. (F) Relative FANCM mRNA expression in U2OS cells treated with different concentrations of gapmer 14. NTC gapmer was used at a concentration of 1 μM. Means ± SD are calculated from at least two biological replicates. (G) Western blot analyses of FANCM levels in cells treated with 1 μM of indicated gapmers for 72 h. β-actin serves as a loading control.

The activity of gapmer 14 was further evaluated in U2OS and LiSa-2 cells and showed a dose-dependent mRNA knockdown with half maximal inhibitory concentration (IC_{50}) around 60 nM (Figure 2F) and 250 nM (Figure S2A), respectively. To correlate mRNA knockdown with protein depletion, FANCM protein levels were measured in ALT cell lines treated for 72 h with 1 μM of gapmers 4, 6, and 14. All three ASOs showed efficient depletion of FANCM protein levels, further confirming the on-target effect of these ASOs (Figure 2G). For LiSa-2 cells, FANCM protein levels after gapmer 14 treatment were assessed in a dose-response manner, showing a reduction in FANCM levels starting at 0.5 μM (Figure S2B).

FANCM ASOs 4, 6, and 14 alter ALT activity

To corroborate FANCM ASOs efficiency with an alteration of ALT activity as previously described,[13,14,26] levels of extrachromosomal telomeric C-circles, a marker of ALT activity,[29] were analyzed in U2OS and SAOS-2 ALT cells treated with gapmers 4, 6, or 14 for 72 h (Figures 3A and 3B). In both cell lines, depletion of FANCM significantly increased levels of telomeric C-circles, as assessed by the C-circle assay. Conversely, treatment of telomerase-positive LPS141 cells with the gapmers did not generate telomeric C-circles (Figure S3A), indicating the specificity of this effect to ALT-positive cancer cells. For LPS141 cells, gapmer concentration was increased to 5 μM instead of 1 μM to ensure a better mRNA knockdown (Figure S3B) and protein depletion (Figure S3C). The effect of gapmer 14 treatment was further evaluated on other phenotypic characteristics in U2OS cells (Figures 3C and 3D). Treating these cells with gapmer 14 led to an increase in the ALT-associated PML bodies (APBs) (Figure 3C), sites of telomeric recombination specifically present in ALT cells,[30] as well as an increase in the percentage of cells with dysfunctional telomeres,[31] assessed by quantifying the number of telomeres colocalizing with the DNA damage response protein 53BP1 (Figure 3D). Collectively, these results indicate that gymnotic delivery of FANCM gapmers is sufficient to alter ALT activity and induce telomeric damage.

FANCM ASOs reduce ALT cell viability *in vitro* and *in vivo*

Given that FANCM ASOs impacted ALT activity, the effect of these treatments was assessed on cell survival potential using clonogenic assays. A single dose of gapmers 4, 6, and 14 at 1 μM reduced the colony formation capacity of the ALT cell lines, U2OS, SAOS-2, and LiSa-2 (Figures 4A and 4B), with LiSa-2 having a lower sensitivity to gapmer treatment in comparison with U2OS and SAOS-2. Conversely, a single treatment with 5 μM of gapmers had varied effects on telomerase-positive cells: MLS402 clonogenic potential was slightly affected, while LPS141 cells were more sensitive to gapmer treatment, especially to gapmer 6 (Figures 4C and 4D). Similar to LPS141, MLS402 was treated with 5 μM instead of 1 μM to increase mRNA and protein depletion (Figures S4A and S4B). These results on survival of telomerase-positive cells are comparable to the effects of FANCM loss seen when using the CRISPR-Cas9 strategy (Figures S1C and S1D). Given that LiSa-2 is our model used for *in vivo* testing in this study, we tested whether increasing the concentration and the number of treatments with FANCM gapmers could further inhibit LiSa-2's survival potential. Since gapmer 14 seemed to have more specific effects to ALT cells compared with gapmer 6 and showed a higher alteration of ALT activity compared with gapmer 4 (Figures 3A and 3B), we tested the response of all the cell lines to a longer and repeated treatment by gapmer 14, using clonogenic assays (Figures 4E and 4F). We also included a control cell line, the normal WI-38 fibroblasts. Here, treating the cells three times every 72 h with 5 μM of gapmers further reduced the number of colonies formed for SAOS-2 and LiSa-2 ALT cells, but not telomerase-positive MLS402 and LPS141 cells, nor WI-38 normal cells (Figures 4E and 4F). FANCM protein reduction in WI-38 after gapmer 14 treatment was verified by western blot (Figure S4C). LPS141 cells were less affected by gapmer 14 in these experiments, compared with the single treatment effect (Figure 4D), potentially due to the longer timeline of these assays, which may have allowed an adaptation response of LPS141 cells to the lack of FANCM.

Next, we tested the capacity of gapmer 14 to inhibit tumor growth *in vivo*. Subcutaneous xenografts of LiSa-2 cells in NOD-SCID mice were treated with either gapmer 14 or a non-targeting control (NTC) gapmer (Figure 4G). Forty milligrams per kilogram of gapmers were administered peritumorally through subcutaneous delivery twice a week. Mice that received gapmer 14 experienced a slight reduction in their body weight mid-treatment, which was recovered by the end of the experiment (Figure S4D). However, no other noticeable symptoms were observed. Importantly, mice receiving gapmer 14 had significantly smaller tumors at the end of the experiment (Figures 4G and 4H) and a lower estimation of tumor growth during the experiment (Figure S4E), demonstrating the anti-tumor efficacy of targeting FANCM with gapmer 14.

DISCUSSION

Despite major advances in our understanding of the alternative lengthening of telomere mechanisms in the past 2 decades, and the discovery of potential targetable vulnerabilities in cancer cells relying on ALT, these cancers still lack clinically approved treatment options. Moreover, preclinical validation of potential ALT-specific targets and therapies remains largely limited due to the absence of adequate *in vivo* models. Here, we validated a previously described ALT target, FANCM, and demonstrated its importance for ALT cell survival *in vivo*, in an ALT-positive liposarcoma xenograft model, LiSa-2, previously reported to form tumors in mice.[32]

As mentioned above, given that no FANCM inhibitor has yet been identified, we opted for the use of antisense oligonucleotides (ASOs) to selectively target FANCM. Here, we identified several gapmers that were effective in down-regulating mRNA and protein levels of FANCM. These gapmers exerted a functional effect on the ALT mechanisms: treating the cells with these ASOs led to telomeric dysfunction, an aberrant ALT activity manifested as an increase in levels of extrachromosomal telomeric C-circle and ALT-associated PML bodies, ultimately resulting in a reduction in cell viability.

Gapmers employing an RNase H1-based cleavage mechanism, such as mipomersen and inotersen, are already approved for clinical use for various diseases.[33] In comparison with unmodified ASOs,

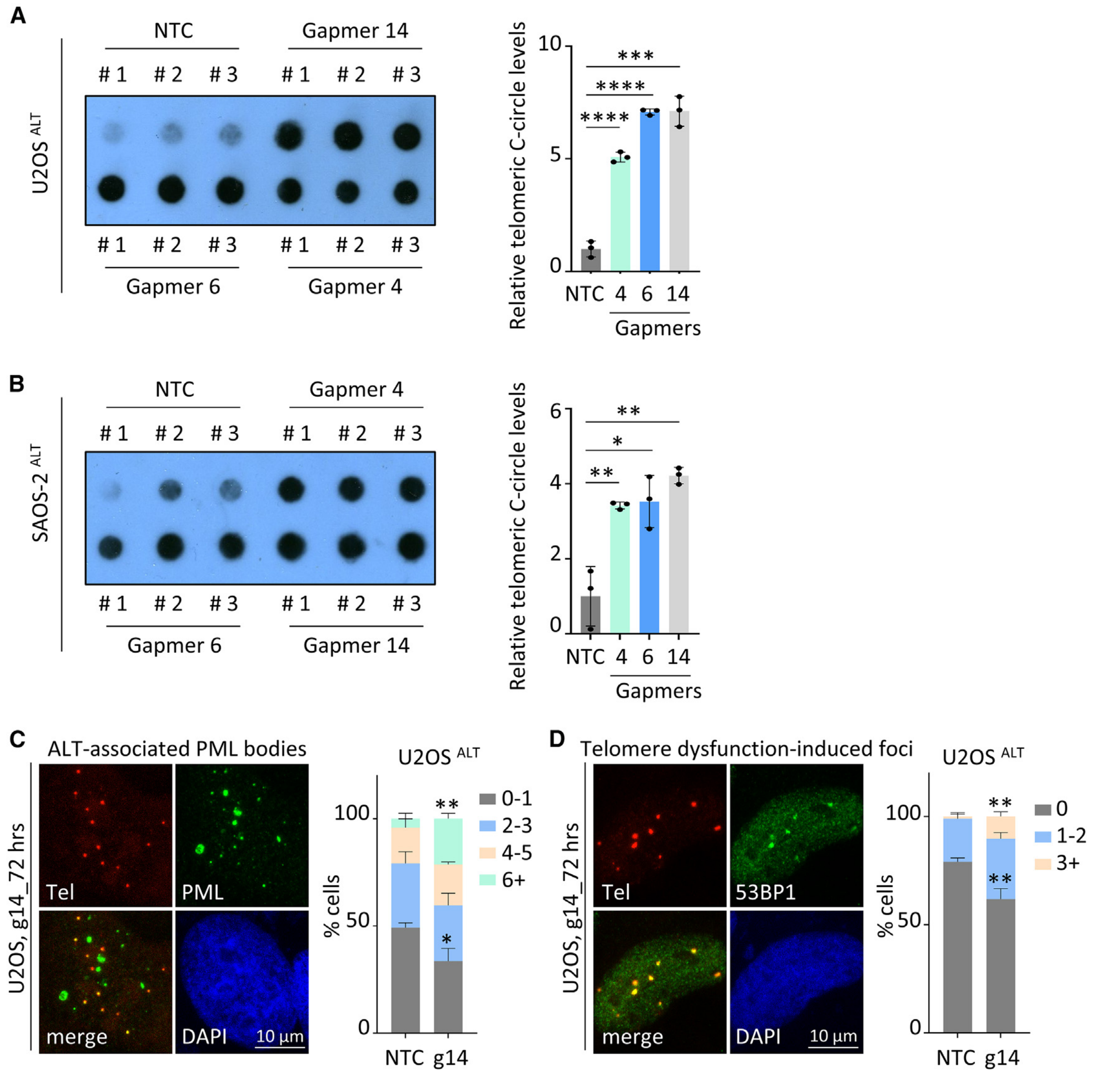

Figure 3. Gapmers 4, 6, and 14 alter ALT activity

(A and B) Telomeric C-circle assays of U2OS (A) or SAOS-2 (B) cells treated with NTC (non-targeting control) gapmer or FANCM-specific gapmers 4, 6, or 14 (at 1 μM for 72 h). Images at the left show three biological replicates for each condition, while graphs represent the quantification (mean ± SD) of C-circle levels relative to those in NTC-treated cells. (C) Quantification of ALT-associated PML bodies (APBs) in U2OS cells treated with NTC or gapmer 14 for 72 h. APBs were scored as co-localization of telomeric (Tel) and PML staining. The graph shows mean percentage of cells (±SD) containing indicated number of APBs. Values are from three biological replicates. (Total number of scored cells: n = 287 and 297 for NTC and gapmer 14, respectively). (D) Telomeric dysfunction-induced foci (TIF) scoring in U2OS cells treated with gapmer 14 for 72 h. Percentages of cells (mean ± SD, from three biological replicates) containing 0, 1–2, or ≥3 TIFs are represented in the graph. (Total number of scored cells: n = 398 and 325 for NTC and gapmer 14, respectively). $^{*}p < 0.05$, $^{**}p < 0.01$, $^{***}p < 0.001$, $^{****}p < 0.0001$ as determined by two-tailed unpaired t test.

gapmers contain chemical modifications that potentiate their antisense activity. For instance, phosphate backbone modifications (phosphorothioate [PS]) in gapmers provide higher resistance to degradation by nucleases, while sugar modifications (locked nucleic acid [LNA]) enhance the binding affinity to the target RNA.[34–36] Moreover, gapmers have the characteristic to be potent at free cellular uptake, and do not require a delivery reagent.[21] To avoid the potential risk of immunogenicity associated with some ASOs,[37,38] our design approach favors gapmers with minimum to no CpG motifs. Except for gapmer 12, which contains one CpG, all the tested gapmers were devoid of CpG stretches, thus reducing risks of adverse immune reactions. The gapmer synthesis protocols used in this study follow standard established protocols used in the community. Therefore, commercially available ASOs should provide similar efficacy to those synthesized here, highlighting the gapmers' production accessibility for potential translational therapies.

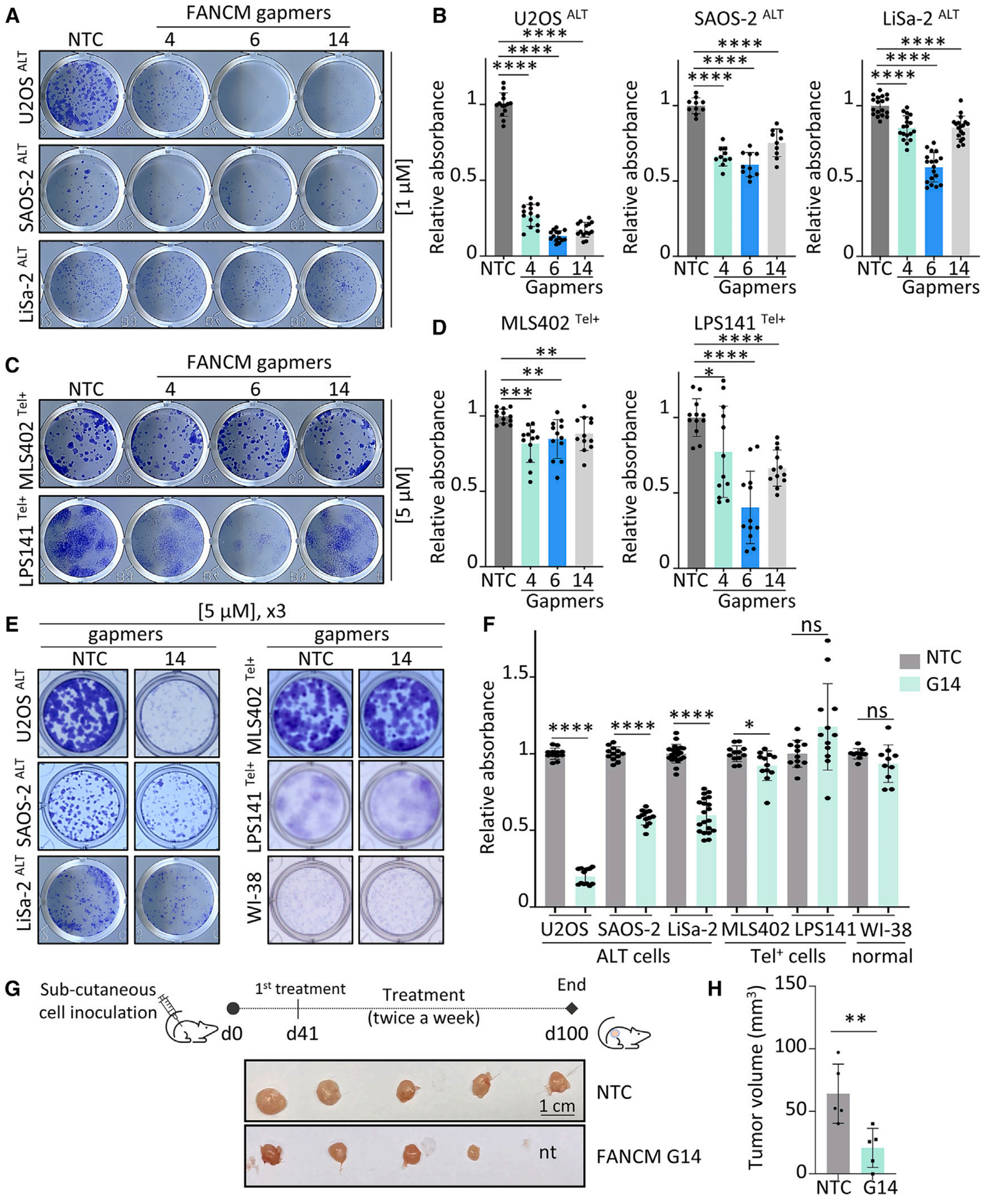

Figure 4. FANCM gapmers 4, 6, and 14 affect ALT cell viability *in vitro* and *in vivo*

(A–F) Colony formation assays on ALT or telomerase-positive cells treated once with 1 or 5 μM of NTC (non-targeting control) or FANCM gapmers (A and C) or three times with 5 μM of gapmers, including normal WI-38 cells (E). Quantification of the absorbance of stained and lyzed colonies is shown in (B), (D), and (F). Values are relative mean (±SD) from biological replicates from two to four independent experiments for each cell line. *$p < 0.05$, **$p < 0.01$, ***$p < 0.001$, ****$p < 0.0001$, ns = not significant, as determined by two-tailed paired t test. (G) Upper panel depicts the *in vivo* experiment timeline for subcutaneous LiSa-2 xenografts treated with either NTC or gapmer 14. Lower panel shows the tumors at the endpoint of the experiment. Each tumor is from one animal (nt = no tumor). (H) Mean volume (±SD) of the tumors shown in (G). **$p < 0.01$ as determined by two-tailed unpaired t test.

Table 1. Forward and reverse sequences for oligonucleotides used for each sgRNA

Gene	Forward oligonucleotide (5′–>3′)	Reverse oligonucleotide (5′–>3′)
FANCM (sgRNA1)	CACCGAATACAACAAGGCATAATCG	AAACCGATTATGCCTTGTTGTATTC
FANCM (sgRNA2)	CACCGTGTCACCAAGGGTTTCGTTG	AAACCAACGAAACCCTTGGTGACAC
HIRA (sgRNA1)	CACCGTGTGTGCGGTGGTCAAACAG	AAACCTGTTTGACCACCGCACACAC
HIRA (sgRNA2)	CACCGAGTCGCAGGCGTTGTCAACG	AAACCGTTGACAACGCCTGCGACTC
ATR (sgRNA1)	CACCGTGACGTGCGAAAACAAGATG	AAACCATCTTGTTTTCGCACGTCAC
ATR (sgRNA2)	CACCGTAGAAGATTAGCGGCAAATG	AAACCATTTGCCGCTAATCTTCTAC

While FANCM depletion seems to affect ALT-positive cell viability more than telomerase-positive cells, the latter were affected, albeit to a lesser extent, by the absence of FANCM. For instance, genetic depletion reduced mildly the viability of two telomerase-positive cells. While the effect is lower compared with that in ALT cells, FANCM may still be important for some telomerase-positive cells. This effect was also seen during gapmer treatments. Telomerase-positive cell viability was affected by gapmer treatment, although mildly, compared with the effect seen with ALT-positive cells. Gapmer 6 had a particularly strong effect on LPS141 cells, potentially indicating an off-target effect, despite our restrictive design strategy, which ensures 100% complementary to only FANCM mRNA. Given the potential off-targets or toxic nature of this gapmer, the more ALT-specific gapmer 14 was chosen for *in vivo* testing.

Gapmer 14 reduced *in vivo* the tumorigenic potential of ALT-positive liposarcoma. The tested concentration was 40 mg/kg, within the range of ASO concentrations used in clinic.[33] At this concentration, no toxicity-related symptoms were seen in the treated animals. The body weight reduction noticed after a few weeks of treatment may have been caused by potential off-targets of the gapmer on mouse genes. This reduction was, however, transient and occurred only after 1 month of beginning the treatment. The animals began to regain weight within 2 weeks, therefore it is also plausible that this body weight reduction may be due to circumstantial experimental inaccuracies. While preclinical testing in mice of efficacy of gapmers as anti-human cancer therapies represent a valuable starting point for assessing potential therapies, late-stage preclinical studies that may include non-human primates may be a suitable model to closely predict the efficacy on cancer cells as well as the effects on normal cells. Given that the potency of gapmer 14 was lower in LiSa-2 cells *in vitro* (IC_{50} ~250 nM compared with ~60 nM for U2OS), we speculate that other models or tumors may respond to a lower concentration. Nevertheless, further *in vivo* investigations will be necessary to study the pharmacokinetic and toxicity profiles of gapmer 14 and to optimize its delivery. For optimal delivery strategy and/or organ-targeted delivery, clinically approved vehicles or direct conjugates may be considered, including the use of nanocarriers, or bioconjugates (e.g., lipids and peptides).[39] Such studies will allow a reduction in the needed dose as well as treatment frequency and potential systemic side effects.

The potential use of FANCM gapmers described in this study is not limited to ALT-positive cancers. Several studies have suggested a synthetic lethality interaction between FANCM and other factors in non-ALT cells, including inhibition of PARP,[40,41] BLM,[42] or WEE1.[43] In addition to cancer therapy, FANCM inhibition using our identified gapmers could be used to improve genome editing *in vitro*.[44]

MATERIALS AND METHODS

Cell culture

Osteosarcoma (OS) cell lines (SAOS-2 and U2OS), WI-38 cells, and HEK293T cells were cultured in Dulbecco's modified Eagle's medium (DMEM, Gibco). Liposarcoma (LPS) cell lines (LiSa-2, LPS141, and MLS402) were grown in Roswell Park Memorial Institute medium 1640 (RPMI, Gibco). The culture media for all cell lines were supplemented with 10% fetal bovine serum (Gibco), 100 units/mL penicillin, and 100 μg/mL streptomycin (Gibco). Cell cultures were maintained in a humidified incubator at 37°C with 5% CO_2 and were free of mycoplasma.

CRISPR-Cas9 plasmid construction

CRISPR-Cas9 plasmids were generated according to the previously described protocol by Sanjana et al.[45] The LentiCRISPRv2puro plasmid (Addgene, # 98290) was digested using the BsmBI restriction enzyme (New England Biolabs) and purified by gel extraction using the QIAquick gel extraction kit (Qiagen). Forward and reverse oligonucleotides specific for each gene were designed[46,47] and purchased from Integrated DNA Technologies (IDT). Two single-guide RNAs (sgRNA1 and 2) were designed for each target. The oligonucleotides were phosphorylated and annealed by incubation for 1 h at 37°C with T4 PNK (New England Biolabs), followed by a 5-min incubation at 95°C and gradual cooling to room temperature. The annealed oligonucleotides and digested plasmid were then ligated using the T4 ligase (New England Biolabs) at 16°C overnight. Bacterial transformation was then conducted using chemically competent Stbl3 bacteria. Colonies were selected, inoculated into 5 mL of LB broth containing ampicillin, and cultivated in a shaking incubator at 37°C for 16 h. Plasmids were then extracted using the FavorPrep Plasmid Extraction Mini Kit (Favorgen) and subjected to sequencing to confirm the insertion of the oligonucleotides. The sgRNA oligonucleotides used are listed in Table 1.

Viral production and cells transduction

Lentiviral particles were generated by transfecting HEK293T cells with 0.25 μg of each of gag/pol (pMDLg/pRRE), Rev (pRSV-Rev), and VSV-G (pMD2.G) plasmids (Addgene, #12251, #12253, and #12259, respectively) and 0.5 μg of the CRISPR plasmid using the

Table 2. Gapmer ASOs used in the study

ASO	Sequence (5′–>3′)	Coordinates of the target region	Region	CpG
Gapmer 1	+T+T+AATATTGTGAG+A+G+G	6,280–6,295	3′UTR	0
Gapmer 2	+T+T+TATCACAACAG+T+A+C	6,568–6,583	3′UTR	0
Gapmer 3	+T+T+AGAATCAGTAC+A+C+T	6,655–6,670	3′UTR	0
Gapmer 4	+T+G+AGATTTTGGTT+G+G+G	6,836–6,851	3′UTR	0
Gapmer 5	+T+C+CTTTTCCACAT+G+G+C	7020–7,035	3′UTR	0
Gapmer 6	+T+G+TGGTCTTGACT+T+G+G	5,489–5,504	exon 21	0
Gapmer 7	+T+C+CTTTCCACCAC+C+A+T	5,683–5,698	exon 21	0
Gapmer 8	+T+T+AAGGTAGTCAG+C+A+G	5,863–5,878	exon 22	0
Gapmer 9	+G+C+TGTTAGCCATC+C+T+T	6,096–6,111	exons 22-23	0
Gapmer 10	+G+T+GGTCTTGACTT+G+G+A	5,488–5,503	exon 21	0
Gapmer 11	+A+T+GTGGTCTTGAC+T+T+G	5,490–5,505	exon 21	0
Gapmer 12	+T+C+AGATATCCGTA+G+C+A	60–75	exon 1	1
Gapmer 13	+T+G+GAAGCTTGTGT+A+G+A	613–628	exon 2	0
Gapmer 14	+T+C+TGGATATAGGT+C+T+T	1,006–1,021	exons 4–5	0
Gapmer 15	+T+T+TAGCATTACTG+C+A+C	5,036–5,051	exon 20	0
Gapmer 16	+C+T+CTGCTTTGCTA+A+T+G	5,279–5,294	exon 20	0
Gapmer 17	+T+T+GACTTGGAACA+G+G+A	5,482–5,497	exon 21	0
Gapmer 18	+T+G+TGTCCCAGCTA+A+A+T	5,504–5,519	exon 21	0
Gapmer 19	+T+C+CTTAGTAACAC+T+T+T	6,489–6,504	3′UTR	0
Gapmer 20	+T+T+TTCTACTTTCC+T+A+G	6,730–6,745	3′UTR	0
Gapmer 21	+G+A+TTTGACAGTAC+T+T+A	6,785–6,800	3′UTR	0
Gapmer 22	+A+A+CCATATCAGAG+T+T+C	6,810–6,825	3′UTR	0
Gapmer 23	+T+T+CAGATTACAAG+T+C+A	6,855–6,870	3′UTR	0
Gapmer 24	+T+C+ATGAGAATATG+T+C+A	935–950	exon 4	0
Gapmer 25	+T+T+TGGATGGCTGC+A+A+G	991–1,006	exon 4	0
Gapmer 26	+T+C+TTGCCAGAATT+A+T+C	1,101–1,116	exon 5	0
Gapmer 27	+T+T+GCTGCAATAAT+T+C+A	1,215–1,230	exon 6	0
Gapmer 28	+T+A+GGTCTTTTGGA+T+G+G	998–1013	exon 4	0
Gapmer 29	+G+A+TATAGGTCTTT+T+G+G	1002–1017	exon 4	0
Gapmer 30	+T+G+GATATAGGTCT+T+T+T	1004–1019	exon 4	0
Non-targeting control (NTC)	+G+G+CTAGATGCTAA+C+C+T	–	–	0

‘+’ indicates LNA monomers. All linkages are phosphorothioate.

PEI 25K transfection reagent (Polysciences). 72 h post-transfection, the supernatant containing lentiviral particles was harvested and filtered using a 0.45-μm syringe filter (Pall Corporation), and subsequently used to infect recipient cells cultured in six-well plates. Media was replaced after 24 h and 1 μg/mL of puromycin (Gibco) was added at 48 h. Infected cells at days 6 or 7 post-selection were collected and used for subsequent experiments.

Design and synthesis of gapmer ASOs

All 16-nt fully phosphorothioated ASOs containing a 3-10-3 gapmer configuration were designed against different regions of FANCM mRNA (RefSeq identifier NM_020937.4). The gapmer ASOs used are in listed in Table 2. These ASOs were produced internally with a DNA/RNA synthesizer (ABI 394) using standard solid phase phosphoramidite chemistry. Controlled pore glass (CPG) supports and DNA and LNA phosphoramidites were purchased from Glen Research. Phenylacetyl disulfide, the sulfurizing agent, was purchased from ChemGenes Corporation. Following the synthesis, the ASOs were cleaved from solid CPG support and deprotected according to the manufacturer’s protocol. The ASOs were purified using reverse-phase HPLC (RP-HPLC), desalted using Glen Pak 2.5 desalting column (Glen Research), and lyophilized. After drying, the ASOs were reconstituted in phosphate-buffered saline (PBS) (Gibco) to a final concentration of 100 μM or 1.2 mM stock solutions before use. Characterization of all ASOs were done using JEOL SpiralTOF MALDI-TOF mass spectrometer.

Table 3. List of quantitative real-time PCR (qRT-PCR) primers

Gene	Forward primer (5′–>3′)	Reverse primer (5′–>3′)
GAPDH	GTCGCCAGCCGAGCCACATC	GGTGACCAGGCGCCCAATACG
FANCM_1	AATCTTGGCTCTAAGTGCCAC	TCTGCCCAATTAGCAGGTTAGTA
FANCM_2	GCCATGCCTCAGGGAAAAG	TAACCACCGTCACGAAACTGT

Gapmer ASO treatment

For all assays, cells were plated 1 day prior to treatment with gapmers. The next day, gapmers were diluted with sterile PBS and added directly to the media to reach the intended final concentration.

Quantitative real-time PCR

ALT-positive cell lines (SAOS-2, U2OS and LiSa-2) and telomerase-positive (Tel+) cell lines (LPS141 and MLS402) were plated at 1×10^5 cells per well and 0.5×10^5 cells per well respectively on six-well plates (Corning) overnight prior to ASOs treatment. Forty-eight hours following gapmer treatment, cells were harvested, and RNA was extracted using EZ-10 DNAaway RNA Mini-Preps Kit (Bio Basic) according to the manufacturer's instructions. cDNA was synthesized using the RevertAid H Minus Reverse Transcriptase (ThermoFisher Scientific) or M-MLV reverse transcriptase (Promega) kits. Synthesized cDNA was diluted to 1:10 and quantitative real-time PCR was performed in Quant Studio 3 (Applied Biosystems) and SYBR Select Master Mix (ThermoFisher Scientific). Relative target expression levels were determined using GAPDH as a housekeeping gene. The primer pairs used are listed in Table 3.

Western blot analysis

Cells were seeded and treated similarly to the procedure for real-time qPCR detailed above. Treated cells were collected 72 h post-ASOs treatment. Total cellular proteins were extracted using RIPA buffer (Thermo Fisher Scientific), supplemented with complete EDTA-free protease inhibitor cocktail tablets (Sigma-Aldrich) and phosphatase inhibitor PhosSTOP tablets (Sigma-Aldrich). Protein concentrations were estimated using Pierce BCA Protein Assay Kit (Thermo Fisher Scientific). Sixty micrograms of proteins were combined with SDS loading buffer, denatured at 95°C for 10 min and then loaded onto an 8% SDS-PAGE gel. Electrophoresis was conducted at 140–160 V for approximately 90 min, followed by a transfer of proteins onto a PVDF membrane (Bio-Rad) at 40 V, overnight at 4°C. Subsequently, the membrane was blocked with 5% blotting-grade blocker (Bio-Rad) in Tris-buffered saline +0.1% Tween 20 (TBST) for 1 h, before incubation overnight at 4°C with primary antibodies (FANCM antibody [CV 5.1] [Novus Biologicals, NBP2-50418]; GAPDH antibody [Cell Signaling Technology, 2118]; ATR [Santa Cruz Biotechnology, sc-515173]; HIRA [Santa Cruz Biotechnology, sc-130636]; ATRX [Santa Cruz Biotechnology, sc-15408]; β-actin [Sigma-Aldrich, A5441]; and β-tubulin [Cell Signaling Technology, 2128]) prepared in 3% blotting-grade blocker in Tris-buffered saline (TBS). Following incubation with primary antibodies, the membrane was washed three times 10 min each with TBST and then incubated with HRP-coupled secondary antibodies for 1 h at room temperature (Rockland antibodies and assays, 18-8817-33 and 18-8816-33; Cell Signaling Technology, 7076), before subsequent TBST washes. The signal was then detected using SuperSignal West Femto Maximum Sensitivity Substrate (Thermo Fisher Scientific) and a ChemiDoc MP imaging system (Bio-Rad) or an Amersham Imager 680.

Telomeric C-circle assay

Cells were plated and treated with gapmers as described above. After 72 h of treatment, genomic DNA was extracted using the DNeasy Blood & Tissue Kit (Qiagen) and quantified with the Qubit 1X dsDNA HS Assay Kit (Invitrogen). Fifty to 75 ng of DNA was then digested using the HinFI and RsaI restriction enzymes (New England Biolabs) for 2 h at 37°C. Rolling circle amplification reactions were afterward performed on 5–7.5 ng of the digested DNA samples using ϕ29 polymerase in ϕ29 buffer, 0.1 mg/mL BSA, and 2 mM dATP, dGTP, and dTTP (New England Biolabs). Amplification was carried out for 6 h at 30°C and terminated at 70°C for 20 min. After amplification, a 5-μL aliquot of the reaction was diluted in saline sodium citrate (2X SSC) and dot blotted onto a Hybond-N+ nylon membrane (GE Healthcare) using a 96-well dotBLOT apparatus (Clever Scientific). Subsequently, the membrane was cross-linked with ultraviolet radiation at 120 mJ, rinsed with 2X SSC, and hybridized with a telomere probe as per the instructions provided by the TeloTAGGG Telomere Length Assay kit (Roche).

Clonogenic assays

Approximately 250 or 500 cells of cells were seeded per well in 24-well plates. For experiments requiring gapmer treatment, ASOs were added after 24 h. Cells were incubated for 9–12 days. Colonies were then fixed with methanol for 10 min and stained with 0.5% (w/v) crystal violet (Sigma-Aldrich) and methanol solution for 20 min. The plates were then rinsed with water and dried overnight at room temperature. Colonies were subsequently lysed using 10% acetic acid, and absorbance was determined at 590 nm using the Tecan Infinite M200Pro plate reader.

Fluorescence *in situ* hybridization and immunostaining

Cells seeded in chamber slides were fixed with formaldehyde (4%) for 10 min, permeabilized for 15 min (PBS +0.5% Triton X-) and blocked for 30 min (in PBS + 3% BSA + 0.1% Triton X-). Slides were then dehydrated with three ethanol washes (50%, 80%, and 100%), before the telomeric PNA probe Cy3-O-O-$(CCCTAA)_3$ (Panagene) (in 70% formamide + 10 mM Tris pH = 7.2 + 1% BSA) was added. Slides were then denatured at 80°C for 5 min and incubated for 2 h at room temperature to allow hybridization. Subsequently, two washes

of each of buffer 1 (70% formamide + 10 mM Tris pH = 7.2) and wash buffer 2 (50 mM Tris pH = 7.2 + 150 mM NaCl + 0.05% Tween 20) were performed for 15 and 5 min, respectively. Slides were then fixed again with formaldehyde (4%) for 5 min, permeabilized for 5 min, and blocked for 30 min. Primary antibodies (PML antibody [Santa Cruz, sc-966] or 53BP1 antibody [Novus biologicals, NB100-304]) diluted in PBS + 1% BSA were added. Following a 1-h incubation at 37°C, slides were washed three times with PBS and re-incubated with fluorescent-labeled secondary antibodies (anti-mouse IgG Alexa Fluor 488 [Life Technologies, A21202] or anti-Rabbit IgG Alexa Fluor 488 [Life Technologies, A11034]) for an hour at 37°C. After PBS washes, slides were mounted with Prolong gold DAPI (Invitrogen) and images were captured using a Zeiss LSM 980 confocal microscope and analyzed using Fiji/ImageJ.

In vivo xenograft experiments

An amount of 1×10^6 LiSa-2 (LacZ sg, FANCM sg1 or FANCM sg2) cells or 2.8×10^6 LiSa-2 cells mixed with Matrigel matrix (Corning) were inoculated subcutaneously in the flank of 6- to 8-week old NOD-SCID (JAX) female mice purchased from InVivos Pte Ltd, Singapore. For the experiment with gapmer treatment, once the tumors were palpable, mice were re-grouped to have a comparable tumor size average between both groups. Control gapmer or FANCM-targeting gapmer 14 (40 mg/kg per mouse) were delivered twice per week by peritumoral injection after dilution in sterile PBS. The weight of mice was followed during the experiment to monitor potential toxicity. At the end of each experiment, mice were euthanized; tumors were collected and measured. *In vivo* experiments were performed in compliance with the ethical regulations of Institutional Animal Care and Use Committee (IACUC) of Nanyang Technological University.

DATA AVAILABILITY

Data generated in this study are available upon reasonable request to the corresponding authors.

ACKNOWLEDGMENTS

This work was funded or supported by: Singapore Ministry of Health's National Medical Research Council (NMRC) (Open Fund—Young Individual Research Grant to M.J. [MOH-00534]), grants from Nanyang Technological University (NTU) to A.T.P., and the NTU PhD scholarship to N.L.B.Y. We are grateful to Fiona Hanindita and Apple Lim Yan Ping for their assistance in the purification of ASOs.

AUTHOR CONTRIBUTIONS

G.T., N.B.Y.L, and M.J. performed experiments and analyzed the data. K.W.L. designed the ASOs. P.D. and A.T.P. contributed to the methodology. M.J. conceptualized the study and supervised the research. G.T., N.B.Y.L., and M.J. wrote the original draft. All authors reviewed and approved the final version.

DECLARATION OF INTERESTS

P.D. is a co-founder and shareholder of LamdaGen Pte. Ltd.

SUPPLEMENTAL INFORMATION

Supplemental information can be found online at https://doi.org/10.1016/j.omtn.2025.102492.

REFERENCES

1. Bryan, T.M., Englezou, A., Dalla-Pozza, L., Dunham, M.A., and Reddel, R.R. (1997). Evidence for an alternative mechanism for maintaining telomere length in human tumors and tumor-derived cell lines. Nat. Med. *3*, 1271–1274. https://doi.org/10.1038/nm1197-1271.
2. Bryan, T.M., Englezou, A., Gupta, J., Bacchetti, S., and Reddel, R.R. (1995). Telomere elongation in immortal human cells without detectable telomerase activity. EMBO J. *14*, 4240–4248. https://doi.org/10.1002/j.1460-2075.1995.tb00098.x.
3. Heaphy, C.M., Subhawong, A.P., Hong, S.-M., Goggins, M.G., Montgomery, E.A., Gabrielson, E., Netto, G.J., Epstein, J.I., Lotan, T.L., Westra, W.H., et al. (2011). Prevalence of the alternative lengthening of telomeres telomere maintenance mechanism in human cancer subtypes. Am. J. Pathol. *179*, 1608–1615. https://doi.org/10.1016/j.ajpath.2011.06.018.
4. Flynn, R.L., Cox, K.E., Jeitany, M., Wakimoto, H., Bryll, A.R., Ganem, N.J., Bersani, F., Pineda, J.R., Suvà, M.L., Benes, C.H., et al. (2015). Alternative lengthening of telomeres renders cancer cells hypersensitive to ATR inhibitors. Science *347*, 273–277. https://doi.org/10.1126/science.1257216.
5. Hoang, S.M., Kaminski, N., Bhargava, R., Barroso-González, J., Lynskey, M.L., García-Expósito, L., Roncaioli, J.L., Wondisford, A.R., Wallace, C.T., Watkins, S.C., et al. (2020). Regulation of ALT-associated homology-directed repair by polyADP-ribosylation. Nat. Struct. Mol. Biol. *27*, 1152–1164. https://doi.org/10.1038/s41594-020-0512-7.
6. Kusuma, F.K., Prabhu, A., Tieo, G., Ahmed, S.M., Dakle, P., Yong, W.K., Pathak, E., Madan, V., Jiang, Y.Y., Tam, W.L., et al. (2023). Signalling inhibition by ponatinib disrupts productive alternative lengthening of telomeres (ALT). Nat. Commun. *14*, 1919. https://doi.org/10.1038/s41467-023-37633-3.
7. Dunham, M.A., Neumann, A.A., Fasching, C.L., and Reddel, R.R. (2000). Telomere maintenance by recombination in human cells. Nat. Genet. *26*, 447–450. https://doi.org/10.1038/82586.
8. Dilley, R.L., Verma, P., Cho, N.W., Winters, H.D., Wondisford, A.R., and Greenberg, R.A. (2016). Break-induced telomere synthesis underlies alternative telomere maintenance. Nature *539*, 54–58. https://doi.org/10.1038/nature20099.
9. Cesare, A.J., Kaul, Z., Cohen, S.B., Napier, C.E., Pickett, H.A., Neumann, A.A., and Reddel, R.R. (2009). Spontaneous occurrence of telomeric DNA damage response in the absence of chromosome fusions. Nat. Struct. Mol. Biol. *16*, 1244–1251. https://doi.org/10.1038/nsmb.1725.
10. Pan, X., Drosopoulos, W.C., Sethi, L., Madireddy, A., Schildkraut, C.L., and Zhang, D. (2017). FANCM, BRCA1, and BLM cooperatively resolve the replication stress at the ALT telomeres. Proc. Natl. Acad. Sci. USA *114*, E5940–E5949. https://doi.org/10.1073/pnas.1708065114.
11. Abbouche, L., Bythell-Douglas, R., and Deans, A.J. (2024). FANCM branchpoint translocase: Master of traverse, reverse and adverse DNA repair. DNA Repair *140*, 103701. https://doi.org/10.1016/j.dnarep.2024.103701.
12. Whitby, M.C. (2010). The FANCM family of DNA helicases/translocases. DNA Repair *9*, 224–236. https://doi.org/10.1016/j.dnarep.2009.12.012.
13. Lu, R., O'Rourke, J.J., Sobinoff, A.P., Allen, J.A.M., Nelson, C.B., Tomlinson, C.G., Lee, M., Reddel, R.R., Deans, A.J., and Pickett, H.A. (2019). The FANCM-BLM-TOP3A-RMI complex suppresses alternative lengthening of telomeres (ALT). Nat. Commun. *10*, 2252. https://doi.org/10.1038/s41467-019-10180-6.
14. Silva, B., Pentz, R., Figueira, A.M., Arora, R., Lee, Y.W., Hodson, C., Wischnewski, H., Deans, A.J., and Azzalin, C.M. (2019). FANCM limits ALT activity by restricting telomeric replication stress induced by deregulated BLM and R-loops. Nat. Commun. *10*, 2253. https://doi.org/10.1038/s41467-019-10179-z.
15. Deans, A.J., and West, S.C. (2009). FANCM connects the genome instability disorders Bloom's Syndrome and Fanconi Anemia. Mol. Cell *36*, 943–953. https://doi.org/10.1016/j.molcel.2009.12.006.
16. Voter, A.F., Manthei, K.A., and Keck, J.L. (2016). A High-Throughput Screening Strategy to Identify Protein-Protein Interaction Inhibitors That Block the Fanconi Anemia DNA Repair Pathway. J. Biomol. Screen *21*, 626–633. https://doi.org/10.1177/1087057116635503.
17. Wu, X., Krishna Sudhakar, H., Alcock, L.J., and Lau, Y.H. (2023). Mannich Base PIP-199 Is a Chemically Unstable Pan-Assay Interference Compound. J. Med. Chem. *66*, 11271–11281. https://doi.org/10.1021/acs.jmedchem.3c00674.

18. Alcock, L.J., Sudhakar, H.K., Young, R., and Lau, Y.H. (2024). Fluorescence polarization assay for screening FANCM-RMI inhibitors to target the alternative lengthening of telomeres. Methods Enzymol. *698*, 361–378. https://doi.org/10.1016/bs.mie.2024.04.014.

19. O'Rourke, J.J., Bythell-Douglas, R., Dunn, E.A., and Deans, A.J. (2019). ALT control, delete: FANCM as an anti-cancer target in Alternative Lengthening of Telomeres. Nucleus *10*, 221–230. https://doi.org/10.1080/19491034.2019.1685246.

20. Dhuri, K., Bechtold, C., Quijano, E., Pham, H., Gupta, A., Vikram, A., and Bahal, R. (2020). Antisense Oligonucleotides: An Emerging Area in Drug Discovery and Development. J. Clin. Med. *9*, 2004. https://doi.org/10.3390/jcm9062004.

21. Stein, C.A., Hansen, J.B., Lai, J., Wu, S., Voskresenskiy, A., Høg, A., Worm, J., Hedtjärn, M., Souleimanian, N., Miller, P., et al. (2010). Efficient gene silencing by delivery of locked nucleic acid antisense oligonucleotides, unassisted by transfection reagents. Nucleic Acids Res. *38*, e3. https://doi.org/10.1093/nar/gkp841.

22. Monia, B.P., Lesnik, E.A., Gonzalez, C., Lima, W.F., McGee, D., Guinosso, C.J., Kawasaki, A.M., Cook, P.D., and Freier, S.M. (1993). Evaluation of 2'-modified oligonucleotides containing 2'-deoxy gaps as antisense inhibitors of gene expression. J. Biol. Chem. *268*, 14514–14522.

23. Crooke, S.T., Lemonidis, K.M., Neilson, L., Griffey, R., Lesnik, E.A., and Monia, B.P. (1995). Kinetic characteristics of Escherichia coli RNase H1: cleavage of various antisense oligonucleotide-RNA duplexes. Biochem. J. *312*, 599–608. https://doi.org/10.1042/bj3120599.

24. Crooke, S.T. (2017). Molecular Mechanisms of Antisense Oligonucleotides. Nucleic Acid Therapeut. *27*, 70–77. https://doi.org/10.1089/nat.2016.0656.

25. Lu, R., and Pickett, H.A. (2022). Telomeric replication stress: the beginning and the end for alternative lengthening of telomeres cancers. Open Biol. *12*, 220011. https://doi.org/10.1098/rsob.220011.

26. Pan, X., Chen, Y., Biju, B., Ahmed, N., Kong, J., Goldenberg, M., Huang, J., Mohan, N., Klosek, S., Parsa, K., et al. (2019). FANCM suppresses DNA replication stress at ALT telomeres by disrupting TERRA R-loops. Sci. Rep. *9*, 19110. https://doi.org/10.1038/s41598-019-55537-5.

27. Khvorova, A., and Watts, J.K. (2017). The chemical evolution of oligonucleotide therapies of clinical utility. Nat. Biotechnol. *35*, 238–248. https://doi.org/10.1038/nbt.3765.

28. Crooke, S.T., Vickers, T.A., and Liang, X.-H. (2020). Phosphorothioate modified oligonucleotide-protein interactions. Nucleic Acids Res. *48*, 5235–5253. https://doi.org/10.1093/nar/gkaa299.

29. Henson, J.D., Cao, Y., Huschtscha, L.I., Chang, A.C., Au, A.Y.M., Pickett, H.A., and Reddel, R.R. (2009). DNA C-circles are specific and quantifiable markers of alternative-lengthening-of-telomeres activity. Nat. Biotechnol. *27*, 1181–1185. https://doi.org/10.1038/nbt.1587.

30. Yeager, T.R., Neumann, A.A., Englezou, A., Huschtscha, L.I., Noble, J.R., and Reddel, R.R. (1999). Telomerase-negative immortalized human cells contain a novel type of promyelocytic leukemia (PML) body. Cancer Res. *59*, 4175–4179.

31. Takai, H., Smogorzewska, A., and de Lange, T. (2003). DNA damage foci at dysfunctional telomeres. Curr. Biol. *13*, 1549–1556. https://doi.org/10.1016/s0960-9822(03)00542-6.

32. Stratford, E.W., Castro, R., Daffinrud, J., Skårn, M., Lauvrak, S., Munthe, E., and Myklebost, O. (2012). Characterization of liposarcoma cell lines for preclinical and biological studies. Sarcoma *2012*, 148614. https://doi.org/10.1155/2012/148614.

33. Egli, M., and Manoharan, M. (2023). Chemistry, structure and function of approved oligonucleotide therapeutics. Nucleic Acids Res. *51*, 2529–2573. https://doi.org/10.1093/nar/gkad067.

34. Eckstein, F. (2014). Phosphorothioates, essential components of therapeutic oligonucleotides. Nucleic Acid Therapeut. *24*, 374–387. https://doi.org/10.1089/nat.2014.0506.

35. Kurreck, J., Wyszko, E., Gillen, C., and Erdmann, V.A. (2002). Design of antisense oligonucleotides stabilized by locked nucleic acids. Nucleic Acids Res. *30*, 1911–1918. https://doi.org/10.1093/nar/30.9.1911.

36. Hagedorn, P.H., Persson, R., Funder, E.D., Albæk, N., Diemer, S.L., Hansen, D.J., Møller, M.R., Papargyri, N., Christiansen, H., Hansen, B.R., et al. (2018). Locked nucleic acid: modality, diversity, and drug discovery. Drug Discov. Today *23*, 101–114. https://doi.org/10.1016/j.drudis.2017.09.018.

37. Volpi, C., Fallarino, F., Pallotta, M.T., Bianchi, R., Vacca, C., Belladonna, M.L., Orabona, C., De Luca, A., Boon, L., Romani, L., et al. (2013). High doses of CpG oligodeoxynucleotides stimulate a tolerogenic TLR9-TRIF pathway. Nat. Commun. *4*, 1852. https://doi.org/10.1038/ncomms2874.

38. Krieg, A.M. (2007). Antiinfective applications of toll-like receptor 9 agonists. Proc. Am. Thorac. Soc. *4*, 289–294. https://doi.org/10.1513/pats.200701-021AW.

39. Roberts, T.C., Langer, R., and Wood, M.J.A. (2020). Advances in oligonucleotide drug delivery. Nat. Rev. Drug Discov. *19*, 673–694. https://doi.org/10.1038/s41573-020-0075-7.

40. Liu, Z., Jiang, H., Lee, S.Y., Kong, N., and Chan, Y.W. (2024). FANCM promotes PARP inhibitor resistance by minimizing ssDNA gap formation and counteracting resection inhibition. Cell Rep. *43*, 114464. https://doi.org/10.1016/j.celrep.2024.114464.

41. Stoepker, C., Faramarz, A., Rooimans, M.A., van Mil, S.E., Balk, J.A., Velleuer, E., Ameziane, N., Te Riele, H., and de Winter, J.P. (2015). DNA helicases FANCM and DDX11 are determinants of PARP inhibitor sensitivity. DNA Repair *26*, 54–64. https://doi.org/10.1016/j.dnarep.2014.12.003.

42. Wang, H., Li, S., Zhang, H., Wang, Y., Hao, S., and Wu, X. (2018). BLM prevents instability of structure-forming DNA sequences at common fragile sites. PLoS Genet. *14*, e1007816. https://doi.org/10.1371/journal.pgen.1007816.

43. Aarts, M., Bajrami, I., Herrera-Abreu, M.T., Elliott, R., Brough, R., Ashworth, A., Lord, C.J., and Turner, N.C. (2015). Functional Genetic Screen Identifies Increased Sensitivity to WEE1 Inhibition in Cells with Defects in Fanconi Anemia and HR Pathways. Mol. Cancer Ther. *14*, 865–876. https://doi.org/10.1158/1535-7163.MCT-14-0845.

44. de Alencastro, G., Puzzo, F., Pavel-Dinu, M., Zhang, F., Pillay, S., Majzoub, K., Tiffany, M., Jang, H., Sheikali, A., Cromer, M.K., et al. (2021). Improved Genome Editing through Inhibition of FANCM and Members of the BTR Dissolvase Complex. Mol. Ther. *29*, 1016–1027. https://doi.org/10.1016/j.ymthe.2020.10.020.

45. Sanjana, N.E., Shalem, O., and Zhang, F. (2014). Improved vectors and genome-wide libraries for CRISPR screening. Nat. Methods *11*, 783–784. https://doi.org/10.1038/nmeth.3047.

46. Doench, J.G., Fusi, N., Sullender, M., Hegde, M., Vaimberg, E.W., Donovan, K.F., Smith, I., Tothova, Z., Wilen, C., Orchard, R., et al. (2016). Optimized sgRNA design to maximize activity and minimize off-target effects of CRISPR-Cas9. Nat. Biotechnol. *34*, 184–191. https://doi.org/10.1038/nbt.3437.

47. Sanson, K.R., Hanna, R.E., Hegde, M., Donovan, K.F., Strand, C., Sullender, M.E., Vaimberg, E.W., Goodale, A., Root, D.E., Piccioni, F., and Doench, J.G. (2018). Optimized libraries for CRISPR-Cas9 genetic screens with multiple modalities. Nat. Commun. *9*, 5416. https://doi.org/10.1038/s41467-018-07901-8.

OMTN, Volume *36*

Supplemental information

Targeting FANCM by antisense

oligonucleotides in ALT-positive cancers

Galen Tieo, Natalie Bao Ying Lim, Kah Wai Lim, Peter Dröge, Anh Tuân Phan, and Maya Jeitany

Figure S1

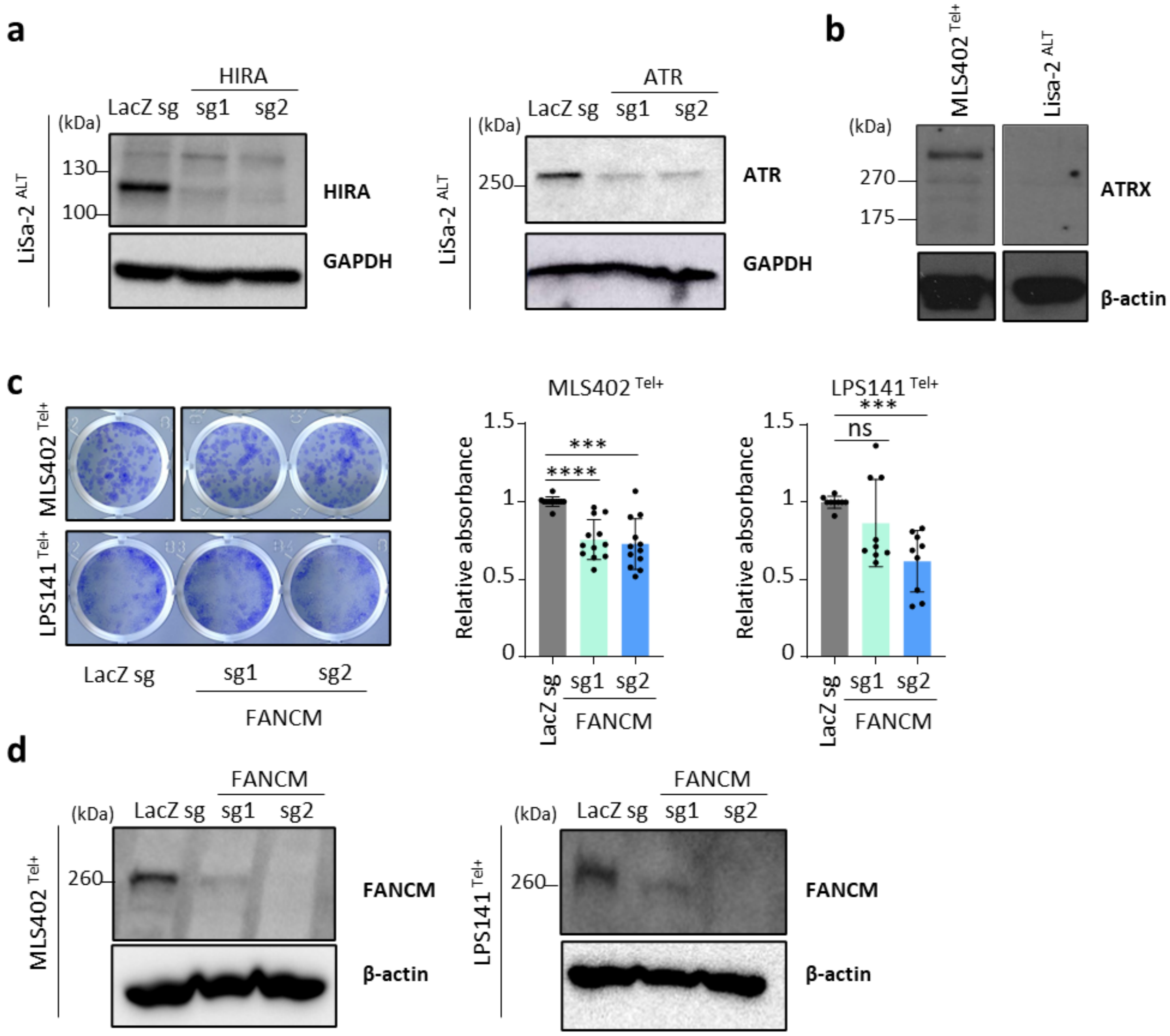

Figure S1: a) Western blots verifying depletion of HIRA or ATR in the corresponding CRISPR-Cas9 edited LiSa-2 cells, compared to LacZ sg control cells. **b)** Western blot showing expression levels of ATRX in MLS402 and LiSa-2 liposarcoma cells. **c)** Colony formation assays for MLS402 or LPS141 telomerase-positive cells, after FANCM depletion. The graphs represent the absorbance quantification (mean ± SD), after staining and lysing of the fixed colonies, relative to corresponding control cells (LacZ sg). Values are from four independent experiments with three biological replicates each for MLS402, and three independent experiments with three biological replicates for LPS141. (***p<0.001, ****p<0.0001, ns = not

significant, as determined by two-tailed paired t-test). **d)** Western blot verifying the reduction of FANCM protein levels in MLS402 and LPS141 FANCM sg1 and sg2 cells. β-actin and GAPDH serve as loading controls.

Figure S2

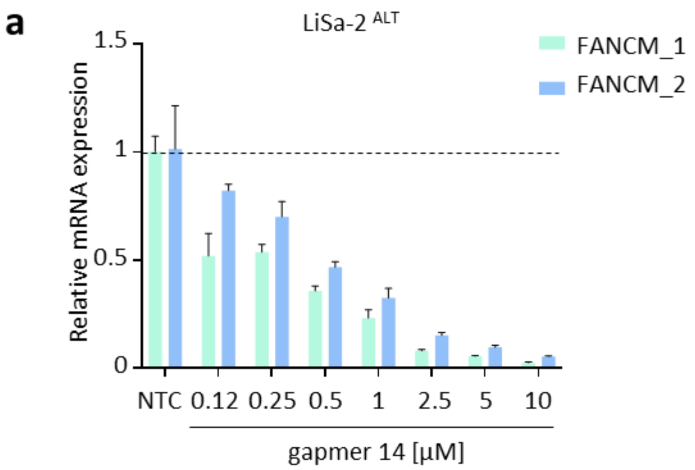

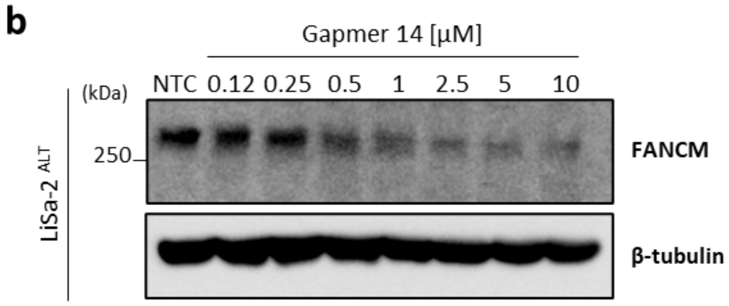

Figure S2: a) FANCM relative mRNA levels detected by qRT-PCR in LiSa-2 cells treated with different concentrations of gapmer 14. NTC gapmer was used at 10 µM. Means ± SD are calculated from three biological replicates. **b)** Western blot assessing FANCM protein levels in LiSa-2 cells treated with similar conditions as in (a). β-tubulin serves as a loading control.

Figure S3

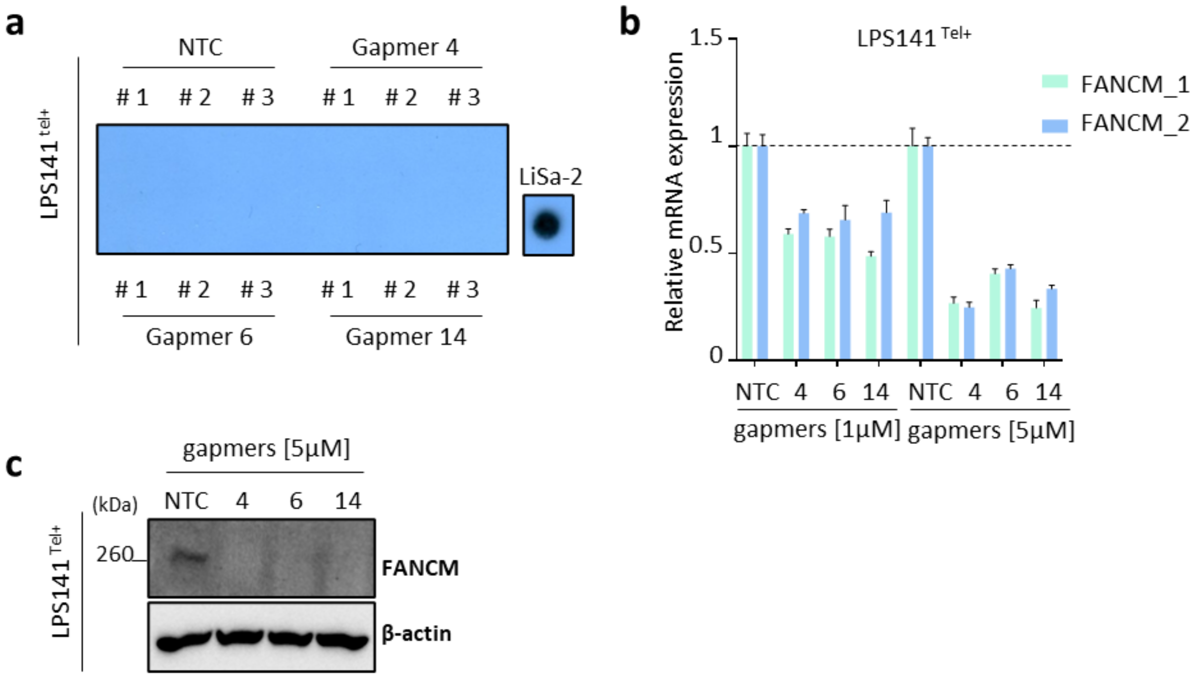

Figure S3: a) Telomeric C-circle assays on LPS141 telomerase-positive cells after 72 hours of treatment with 5 µM of control (NTC) or FANCM-targeting gapmers 4, 6 or 14 (three biological replicates each). LiSa-2 is shown as a positive control. **b)** FANCM mRNA levels detected by qRT-PCR in LPS141 treated with either 1 or 5 µM of indicated gapmers. Values (mean ± SD) are shown relative to NTC control and are calculated from three biological replicates. **c)** Western blot detecting FANCM in LPS141 treated with gapmers for 72 hours. β-actin is a loading control.

Figure S4

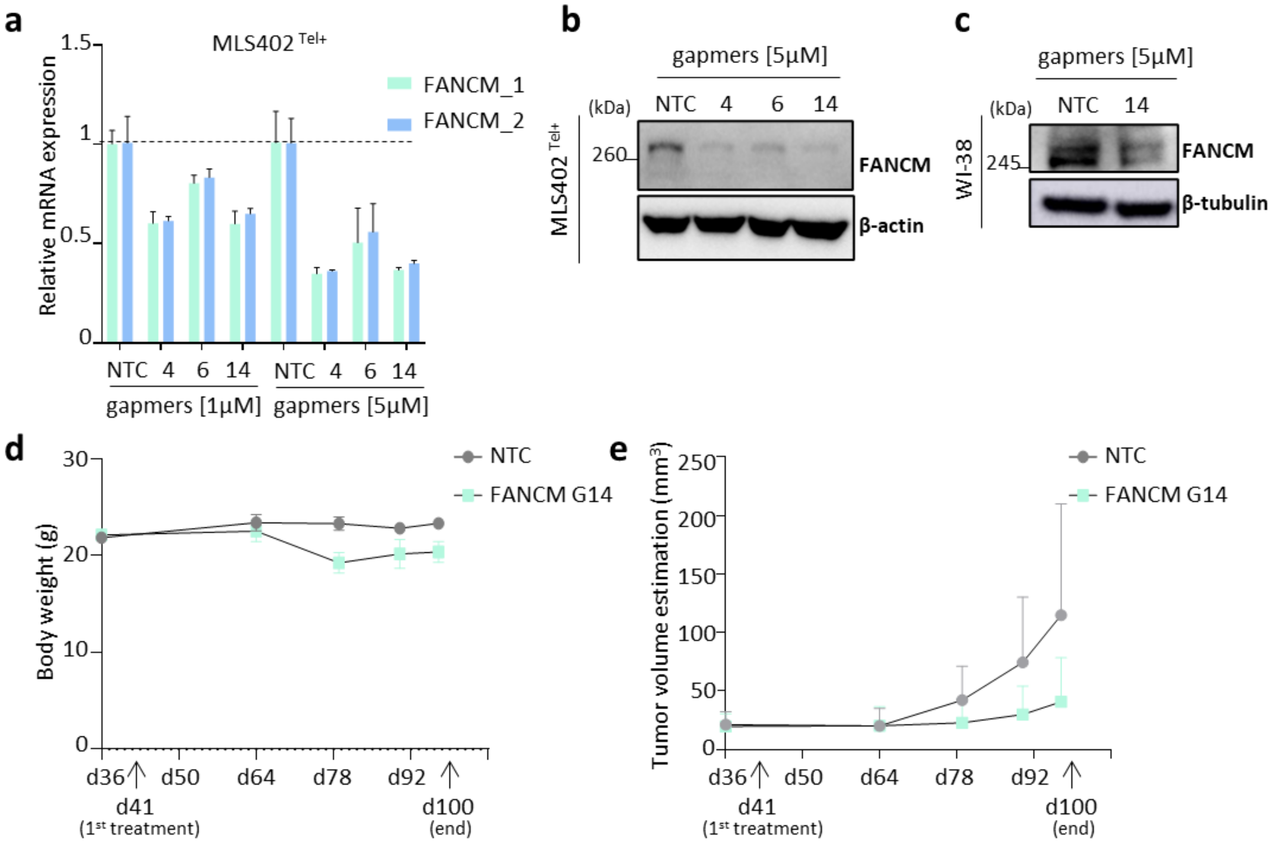

Figure S4: a) qRT-PCR detection of FANCM expression in MLS402 cells treated with indicated gapmers for 48 hours. Relative mean (± SD) of three biological replicates is shown for each condition. **b-c)** Western blot analyses showing reduction of FANCM protein levels in MLS402 cells treated with 5 µM of gapmers 4, 6 or 14 for 72 hours (b), and in WI-38 cells treated with gapmer 14 for 72 hours (c). β-actin serves as a loading control. **d-e)** Follow-up of body weight (d) and tumor volume (e) of mice from the experiment in 4g-h. Values are mean (± SD) body weight of all animals (d) or tumor volumes (e).
